# Launch of a Nationwide Hepatitis C Elimination Program — Georgia, April 2015

**DOI:** 10.15585/mmwr.mm6428a2

**Published:** 2015-07-24

**Authors:** Kiren Mitruka, Tengiz Tsertsvadze, Maia Butsashvili, Amiran Gamkrelidze, Paata Sabelashvili, Ekaterine Adamia, Mari Chokheli, Jan Drobeniuc, Liesl Hagan, Aaron M. Harris, Tea Jiqia, Ana Kasradze, Stephen Ko, Vakhtang Qerashvili, Lali Sharvadze, Irina Tskhomelidze, Valeri Kvaratskhelia, Juliette Morgan, John W. Ward, Francisco Averhoff

**Affiliations:** 1 Division of Viral Hepatitis, National Center for HIV/AIDS, Viral Hepatitis, STD, and TB Prevention, CDC; 2 Infection Diseases, AIDS, and Clinical Immunology Research Center, Tbilisi, Georgia; 3 Neolab, Tbilisi, Georgia; 4 National Center for Disease Control and Public Health of Georgia, Ministry of Labor Health and Social Affairs of Georgia; 5 Georgian Harm Reduction Network, Tbilisi, Georgia; 6 Ministry of Labor Health and Social Affairs of Georgia; 7 Open Society Foundation, Tbilisi, Georgia; 8 CDC Foundation; 9 State Regulation Agency for Medical Activities, Ministry of Labor Health and Social Affairs of Georgia; 10 Boston University School of Public Health; 11 Georgian French Joint Hepatology Clinic, Tbilisi, Georgia; 12 Global Disease Detection, Division of Global Health Protection, South Caucasus CDC Office, Tbilisi, Georgia

Hepatitis C virus (HCV) infects an estimated 130–150 million persons globally and results in an estimated 700,000 deaths annually from hepatocellular carcinoma or cirrhosis (1,2). Georgia, a middle-income Eurasian country, has one of the highest estimated HCV prevalences in the world (3). In 2011, Georgia began offering treatment to a limited number of HCV-infected persons. Beginning in 2013, when new oral medications that can cure >90% of HCV infections were licensed (4,5), Georgia engaged partners to develop a comprehensive HCV prevention and control plan, during which the concept of elimination of HCV transmission and disease emerged. To prepare for the launch of an HCV elimination program, Georgia requested CDC’s assistance to describe HCV epidemiology, evaluate laboratory and health care capacity, and conduct program monitoring and evaluation. This report describes the activities undertaken to prepare for the program, launched in April 2015, and early results of its initial phase, focused on improving access to affordable diagnostics and free curative treatment for HCV-infected persons with severe liver disease. A national population-based serosurvey began in May 2015, and four clinical sites and their laboratories were selected as initial pilot sites; since June, three additional sites have been added. Through July 3, 2015, a total of 6,491 persons sought treatment, and 6,177 (95.2%) initiated diagnostic work-up. Among these, 1,519 (24.6%) completed work-up, 1,474 (97.0%) of whom initiated treatment. Georgia is scaling up capacity to meet the demand for HCV treatment and is collaborating with CDC and other partners on development of a comprehensive HCV elimination plan that includes specific goals and activities needed to achieve them.

Based on the finding of 6.7% anti-HCV seroprevalence in a survey in Tbilisi, Georgia’s capital and largest city, in 2002 (3), an estimated 250,000 persons among the country’s 3.7 million inhabitants might be infected with HCV. Injection drug use is a major risk factor for HCV infection (3), although unsafe injection and blood safety practices also contribute to the infection burden (6). The prevalence of HCV infection is high among prisoners (50%) (Georgia’s Ministry of Labor, Health, and Social Affairs [MoLHSA], unpublished data, 2015), injection drug users (50%–70%) (7), and persons infected with human immunodeficiency virus (HIV) (47%) (8).

Anti-HCV serologic testing is widely available in Georgia. However, tests for RNA to identify active infection, genotyping to determine strain, and fibrosis staging to assess severity of liver disease (all necessary for clinical decision-making) are expensive and more difficult to obtain. Georgia’s universal health care system requires most persons to pay out-of-pocket for HCV diagnosis and treatment, resulting in treatment of only 100–150 patients annually, before 2011 (MoLHSA, unpublished data, 2015). In 2011, Georgia implemented programs to increase access to HCV treatment with pegylated interferon and ribavirin (PEG/RBV) among HIV-coinfected persons, prisoners, and the general population (Table), which has resulted in approximately 1,685 Georgians receiving treatment to date.

In 2013, the government of Georgia requested technical assistance from CDC to develop a comprehensive HCV prevention and control strategy. CDC, MoLHSA, and other national and international partners met in 2014 and identified a national HCV seroprevalence survey and improved access to new curative HCV treatment as initial priorities. The potential for HCV elimination in Georgia was recognized on the basis of the absence of a nonhuman viral host, available effective diagnostics, prevention, and treatment (9,10), and the country’s small size and population, experience with HIV prevention and control programs, strong political will, and public support. Georgia committed to building its capacity to implement an HCV elimination program. of ledipasvir-sofosbuvir (Harvoni) annually at no cost. The HCV elimination program was to be initially focused on treating HCV-infected persons with severe liver disease and providing discounted HCV diagnostic services. Georgia requested assistance from CDC to 1) conduct a national survey to define epidemiology and disease burden, 2) assess laboratories and health care providers to identify sites with capacity to participate in the initial phase of the elimination program, and 3) monitor and evaluate the program (Table).

A stratified, multistage cluster survey designed to select a nationally representative sample of 7,000 adults, calculated based on current HCV prevalence estimates and an anticipated 70% response rate, was launched in six major cities (including Tbilisi) and 10 rural regions in May 2015. Serum samples for anti-HCV antibody (and, if positive, HCV RNA and genotyping) and data on behavioral risk factors are collected during household visits. The survey will allow calculation of independent HCV prevalence estimates for the six major cities and most rural areas surveyed once analyzed by fall 2015.

Eight clinical sites and two prisons with experience providing interferon-based treatment were assessed and scored based on six domains: leadership and governance, quality of clinical care services, health information systems/management, human resource capacity, access to necessary laboratory tests, and drug-procurement procedures. A standard World Health Organization–adapted tool was used to assess capacity at four clinical laboratories (affiliated with some of the assessed clinical sites) and eight public health laboratories regarding biosafety, specimen collection and accessioning, equipment and test kit use, staff competency, quality assurance and quality control (QA/QC), and reporting and communication.*

A data management system (STOP-C) was developed to collect demographic, diagnostic, clinical, and pharmacy data on patients registered for treatment, which permits data entry by health care providers as well as the Central Social Service Agency (based at MoLHSA in Tbilisi). CDC provided technical support in identifying key variables for monitoring the HCV continuum of care.

Four of the highest scoring clinical sites in Tbilisi and their corresponding laboratories were selected as initial pilot sites for the elimination program. All four laboratories provide point-of-care and laboratory-based anti-HCV testing, viral load determination, and genotyping. Although one of the laboratories had International Organization for Standardization 15189 medical laboratory certification,† which specifies requirements for quality and competence in medical laboratories, all lacked external QA/QC procedures, and efforts are underway to develop such a program and validate test kits. The health care provider assessment revealed limited experience with the new HCV medications and a need for additional training and case management support. Since June 2015, three additional clinical sites with moderate scores and their laboratories in two other cities have been added to meet demands for HCV diagnosis and treatment; improvement in capacity is ongoing at these sites.

An HCV Elimination Program Treatment Inclusion Committee, consisting of clinicians, patient advocacy representatives, and media, was established to review each (de-identified) patient record to determine treatment eligibility and appropriateness of provider-recommended regimens, and to ensure transparency and equitability of access to treatment. As of July 3, 2015, among 6,491 HCV-positive persons who sought treatment, 6,177 (95.2%) initiated diagnostic work-up, of whom 1,519 (24.6%) had completed evaluation and obtained required documentation for treatment consideration. The committee has evaluated and approved 1,474 (97.0%) of these patients for treatment initiation, and all 1,474 have started treatment.

## Discussion

The response to the initial phase of Georgia’s HCV control program has been larger than that for earlier PEG/RBV access programs. Increased demand likely is the result of the availability of free, effective, well-tolerated, and curative treatment options, coupled with affordable diagnostics for HCV-infected persons with advanced liver disease, who are at greatest risk for morbidity and mortality. Additional provider training and case management support are remaining challenges. MoLHSA initially limited the number of participating sites, to ensure quality and appropriate clinical decision making; the recent addition of three new sites should reduce program delays and facilitate program expansion, and assessment of additional providers and laboratories is ongoing. Monitoring and evaluation will continue, and efforts are ongoing to develop an external QA/QC system to be used by laboratories to achieve and maintain biologic safety and quality diagnostic standards.

Although HCV is a strong candidate for elimination in Georgia, many challenges exist, including the asymptomatic, chronic nature of disease, which results in diagnostic delays, and ongoing transmission in health care settings and among hard-to-reach populations (e.g., injection drug users) with potential for reinfection. To address these challenges, Georgia is developing a comprehensive elimination plan that addresses advocacy and communication, surveillance (including quality diagnostics), prevention (e.g., infection control, blood safety, and harm reduction), and testing and linkage to care.§ An international technical advisory committee is being formed to help define achievable and measurable elimination goals and indicators, and determine priority activities. Additionally, MoLHSA has begun to implement broader HCV control activities, including a campaign to raise awareness, provision of free HCV testing to identify HCV-infected persons unaware of their infection status, and improved infection control practices. Georgia’s elimination program can provide information and experience that will assist similar efforts in other parts of the world.

**Summary**

What is already known on the topic?

Hepatitis C virus (HCV) infection is a serious health problem that affects an estimated 130–170 million persons globally and results in an estimated 700,000 deaths annually. In 2013, new all-oral, well-tolerated regimens were licensed that can cure >90% of HCV infections. The country of Georgia has one of the world’s highest estimated HCV prevalences.

What is added by this report?

In April 2015, Georgia launched a hepatitis C elimination program that will initially focus on treating HCV-infected persons who have severe liver disease with new curative regimens, providing discounted HCV diagnostics to all persons, and building capacity to eventually diagnose and treat all Georgians infected with HCV. A national serosurvey was launched in May 2015, and seven clinical sites have opened to diagnose and treat HCV. Georgia is scaling up capacity to meet the high demand for HCV treatment.

What are the implications for public health practice?

Georgia has increased access to HCV testing and treatment as part of preparatory phase of a national HCV control program with goals for the elimination of HCV transmission and disease in the country. Georgia’s program can provide information and experience that will assist similar efforts in other parts of the world.

## Figures and Tables

**TABLE:** Key strategies, activities, and outcomes before implementation of a nationwide hepatitis C elimination program — Georgia, 2011–2015

Strategy	Period	Activity	Outcome
Improve treatment access	2011–present	Free PEG/RBV treatment for up to 110 HIV/HCV co-infected persons per year through Global Fund for AIDS, TB, and Malaria	428 persons received treatment*
	2014–present	Free HCV screening, diagnostics for all incarcerated persons Free PEG/RBV treatment for up to 500 incarcerated persons with fibrosis stage ≥F2 (moderate disease) per year	406 persons received treatment*
	2014–present	Reduced price (60%) PEG/RBV treatment for 10,000 persons	851 persons received treatment*
	2015	5,000 free courses of sofosbuvir (Sovaldi), followed by 20,000 free courses of ledipasvir-sofosbuvir (Harvoni) per year through Gilead Science	1,474 persons received treatment†
Secure political commitment	2014	Georgian government prioritizes hepatitis C control	Establishment of national HCV commission
Partnership development	2013–2015	Engagement of international public health, academic, and industry partners to strengthen HCV response, with goal of elimination	CDC technical support Commitment from Gilead to provide free new curative medications
Capacity assessment	2015	Assessment of four clinical and eight public health laboratories	Development of test validation panels Recommendation for QA/QC plan
		Assessment of eight clinical sites and two prisons	Identification of first elimination program sites (i.e., total of seven sites to date, including four initial pilot sites in Tbilisi) Identification of critical gaps
National planning	2015	Definition steps for the initial phase of elimination program (key activities and treatment protocols)	Approval of initial activities and treatment protocols
Monitoring and evaluation	2015	Expanded data system used to track care and treatment during interferon access program	Development of STOP-C data management system to monitor and evaluate HCV continuum of care
Provider education	2015	Training of providers in HCV management	Ongoing
Defining disease burden	2015	National seroprevalence survey	Ongoing
Raising awareness	2015	Public campaign “STOP-C” developed by Georgia’s Ministry of Labor, Health, and Social Affairs and partners to raise awareness for diagnosis and treatment of hepatitis C	Ongoing

**Abbreviations:** PEG/RBV = pegylated interferon and ribavirin; HCV = hepatitis C virus; HIV = human immunodeficiency virus; QA/QC = quality assurance and quality control.

* As of July 2015.

† During April 28–July 3, 2015.

## Footnotes

* Additional information available at http://www.who.int/ihr/publications/laboratory_tool.

† Additional information available at http://www.iso.org/iso/catalogue_detail?csnumber=56115.

§ Additional information available at http://www.who.int/csr/disease/hepatitis/GHP_framework.pdf.
